# Preclinical Evaluation of *Tradescantia spathacea* Phenolic Extract-Loaded Silica in a Parkinson’s Disease Model

**DOI:** 10.3390/molecules31060950

**Published:** 2026-03-12

**Authors:** Lorenna E. S. Lopes, Marília R. Oliveira, Reinaldo V. B. Neto, Tatiane B. Santos, Juliana F. De Conto, M. Beatriz P. P. Oliveira, Margarete Z. Gomes, Klebson S. Santos

**Affiliations:** 1 Graduate Program in Health and Environment, Tiradentes University (UNIT), Av. Murilo Dantas, 300, Aracaju 49032-490, Sergipe, Brazil; lorenna.sena@souunit.com.br (L.E.S.L.); reinaldo.viana@souunit.com.br (R.V.B.N.); tatiane.bdos@souunit.com.br (T.B.S.); 2 Center for Study on Colloidal Systems (NUESC), Institute of Technology and Research (ITP), Av. Murilo Dantas, 300, Aracaju 49032-490, Sergipe, Brazil; marilia_santos@itp.org.br (M.R.O.); juliana_faccin@itp.org.br (J.F.D.C.); 3 Postgraduate Program in Process Engineering (PEP), Tiradentes University (UNIT), Av. Murilo Dantas, 300, Aracaju 49032-490, Sergipe, Brazil; 4 REQUIMTE/LAQV, Laboratory of Bromatolgy, Department of Chemical Sciences, Faculty of Pharmacy, University of Porto, R. Jorge de Viterbo Ferreira, 228, 4050-313 Porto, Portugal; beatoliv@ff.up.pt

**Keywords:** administration intranasal, *commelinaceae*, Parkinson’s disease, polyphenols

## Abstract

The current limitations in Parkinson’s Disease (PD) treatments necessitate innovative approaches. To this end, phenolic compounds from *Tradescantia spathacea* (*T. spathacea*) and bioactive silica demonstrate potential therapeutic efficacy in the prevention or treatment of neurodegenerative disorders, including Alzheimer’s disease and Parkinson’s disease. Hence, this study explores the neuroprotective potential of silica loaded with *T. spathacea* extract (SiO_2_-TS) in a preclinical model of PD. The aqueous extract of *T. spathacea* (AETS) was prepared via infusion and characterized in terms of overall yield (21.9 ± 0.4%), total phenolic compounds (25.51 ± 2.39 mg GAE/g), and total flavonoid content (6.10 ± 0.16 mg RE/g). Silica loaded with AETS was synthesized and tested in adult Wistar rats (PD-like symptoms). The rats were treated with daily intranasal administration of SiO_2_-TS (10 or 30 mg/kg) for 15 days. Quantitative behavioral analysis showed significant motor improvement and reduced anxiety-like behavior in the 30 mg/kg SiO_2_-TS group compared to the 6-OHDA (6-hydroxydopamine) control. Immunohistochemistry revealed preserved dopaminergic neurons and reduced astrogliosis (GFAP expression) in the same SiO_2_-TS group. These results suggest SiO_2_-TS has significant neuroprotective effects and warrants further study for Parkinson’s disease treatment.

## 1. Introduction

Population aging represents a global public health challenge, primarily due to Alzheimer’s and Parkinson’s disease (PD). PD is the second most prevalent neurodegenerative disorder worldwide [1,2]. It is characterized by the degeneration of dopaminergic neurons in the substantia nigra pars compacta (SNpc), leading to a reduction in dopamine levels at both pre-and postsynaptic terminals in the dorsal striatum [3]. This deficiency results in motor symptoms such as bradykinesia, postural instability, resting tremor, muscle rigidity, dysphagia, dysarthria, and sensory disturbances [4].

Neuronal death in PD is known to be associated with cellular events such as neuroinflammation and oxidative stress. Neuroinflammation triggers a cascade of deleterious effects on dopaminergic neurons, involving the abnormal migration of glial cells, particularly astrocytes and microglia [5]. Dysregulated astrocytic responses interfere with neuronal structural and metabolic support, synaptic transmission, neurotrophic molecule production, blood–brain barrier maintenance, and assistance to microglia in immune responses [6].

Additionally, nitric oxide synthase—activated by the neuroinflammatory cascade—contributes to neuronal death through the production of hydroxyl radicals and reactive oxygen species (ROS), such as peroxynitrite [7]. Increased ROS levels, due to an imbalance in cellular redox potential, can lead to neuronal death and dopamine oxidation [8]. Given that heightened neuronal metabolic activity generates greater ROS production, and that the SNpc region contains a high concentration of oxidizable species, this area is particularly vulnerable due to its extensive metabolic activity, abundant dopamine content, and low levels of endogenous antioxidants like glutathione [9].

The standard treatment for PD symptoms is L-3,4-dihydroxyphenylalanine, known as Levodopa, which primarily alleviates motor symptoms [10]. Although effective, approximately 80% of patients develop adverse effects—such as dyskinesias and motor fluctuations—during the advanced stages of the disease [11]. Additionally, Levodopa exhibits low bioavailability following oral administration and limited brain uptake [12].

Owing to the limitations previously mentioned, the development of new therapeutic approaches is essential. Exogenous antioxidants derived from plant extracts may offer a promising alternative. *T. spathacea*, commonly known as purple pineapple, is a herbaceous plant from the Commelinaceae family, endemic to Central America and easy to cultivate [13]. Its chemical composition includes various bioactive compounds with antioxidant, anti-inflammatory, and neuroprotective properties, such as phenolic acids, flavonoids, coumarins, and iridoids [14]. The antioxidant activity of *T. spathacea* bioactive compounds is considered high, showing free radical scavenging capacity comparable to tocopherol and superior to ascorbic acid [13].

Drug delivery systems may offer a viable strategy for administering bioactive compounds effectively. Silica-based materials are particularly promising due to their porous structures, which allow for high-capacity adsorption or encapsulation of biocompounds [15]. These silica particles exhibit characteristics such as extensive surface area, large pore volume, and adjustable pore size, making them well-suited for carrier platform development [16].

Since neuronal death in PD is linked to neuroinflammation and oxidative stress [5], and considering that *T. spathacea* extract has demonstrated neuroprotective effects while reducing astrocytic reactivity [14], and that silica particles are effective biocompound carriers [17], intranasal delivery of silica particles loaded with *T. spathacea* extract may represent an innovative neuroprotective approach for PD. The interaction between natural compounds and bioactive materials to optimize the delivery of bioactive agents remains underexplored. Hence, this study aims to synthesize mesoporous silica loaded with *T. spathacea* extract and evaluate its neuroprotective effect and modulation of astrocyte reactivity following intranasal administration in a preclinical model of Parkinson’s Disease, aiming to achieve efficacy with minimal sedation.

## 2. Results

### 2.1. Extraction Yield and Total Polyphenols of AETS

The AETS exhibited an overall extraction yield of 21.9 ± 0.4%, indicating efficient recovery of soluble bioactive constituents. Phytochemical analysis quantified a total phenolic content (TPC) of 25.51 ± 2.39 mg GAE/g extract), suggesting a significant presence of phenolic compounds, which are well-established for their antioxidant and potential neuroprotective bioactivities. Furthermore, spectrophotometric assays revealed a total flavonoid content (TFC) of 6.10 ± 0.16 mg RE/g extract, highlighting the contribution of flavonoid secondary metabolites to the extract’s pharmacological potential. The quantified levels of polyphenolic compounds within AETS warrant further investigation into its bioactivity, particularly in the context of neurodegenerative disorders.

### 2.2. Characterization of Silica

#### 2.2.1. Thermogravimetric Analysis

**Figure 1:**
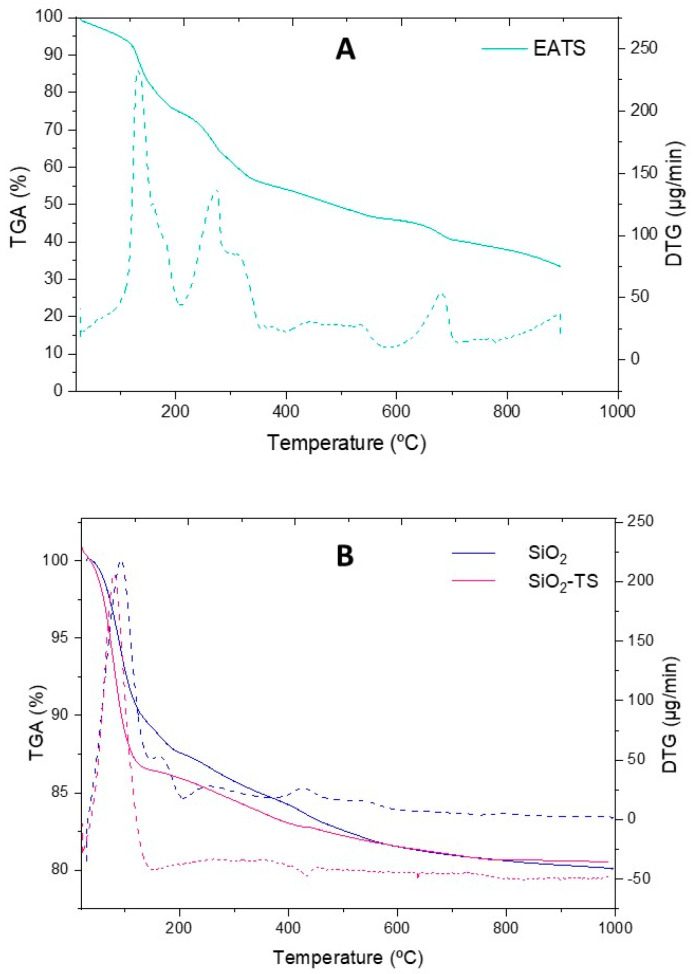
Thermogravimetric analysis of the AETS sample (**A**) and the SiO_2_ and SiO_2_-TS sample (**B**). Expressed as percentage of mass lost (%TG—solid line) and percentage of the derivative per minute (DTG %/min—dashed line).

The results (Figure 1) indicated that the samples containing silica (SiO_2_ and SiO_2_-TS) exhibited greater thermal stability, losing less mass over time and at the pre-established temperatures compared to the AETS. Mass loss in all samples began at approximately 50 °C, likely associated with the release of water and the degradation of heat-sensitive biocompounds. The AETS showed greater instability, with significant degradation occurring at several points, resulting in a total mass loss of 72.5% during the analysis. The initial mass loss, observed between 100 °C and 120 °C, is attributed to compound dehydration. The second mass loss, occurring between 120 °C and 200 °C, is related to the melting of low molecular weight organic compounds such as flavonoids, isoflavonoids, and other phenolic compounds. Finally, the most substantial mass loss, between 220 °C and 450 °C, is attributed to the decarboxylation of the remaining organic material [18].

The SiO_2_ sample exhibited a total mass loss of 19.8% throughout the analysis, with the most pronounced loss occurring at approximately 100 °C (10.8%), attributed to water adsorbed within the pores of the silica particle in an endothermic reaction [19]. Subsequently, discrete mass losses were noted at 150, 250, 425, 545, and 795 °C, which together accounted for an additional 9% mass loss. These events are mainly attributed to the progressive condensation of surface silanol groups (Si–OH), leading to the formation of siloxane bonds (Si–O–Si) with the release of water molecules, a process typically observed in sol–gel derived silica materials [19,20].

The plant extract carrier particles (SiO_2_-TS) showed a lower total mass loss of only 14.5% throughout the entire analysis. Between 50 and 150 °C, a mass loss of 11.8% was observed, attributed to the presence of water. Shortly thereafter, slight mass losses of 1.9% and 0.8% occurred at temperatures of 200–250 °C and 400 °C, respectively, corresponding to the plant extract incorporated into the silica.

#### 2.2.2. Fourier Transform Infrared Spectroscopy

**Figure 2:**
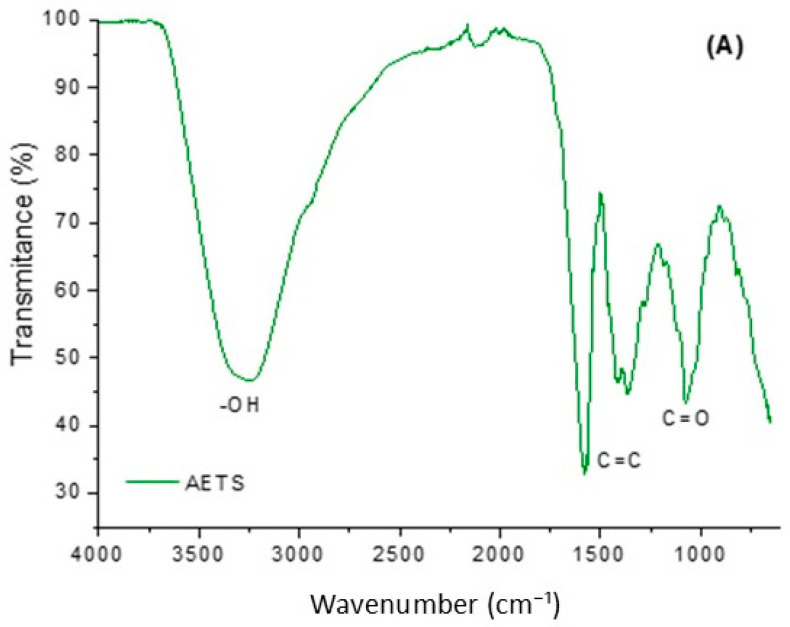

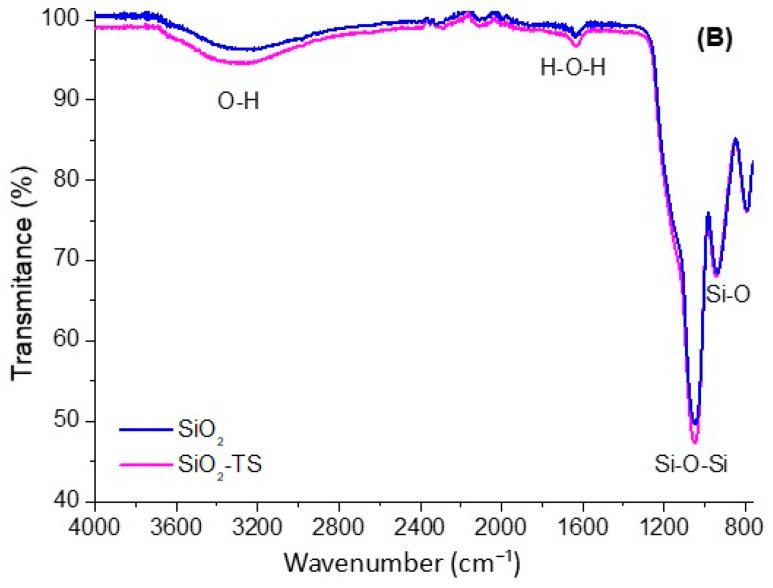
FTIR spectra of the AETS sample (**A**) and the SiO_2_ and SiO_2_-TS samples (**B**).

FTIR was performed to verify the presence of functional groups on the surface of AETS, SiO_2_, and SiO_2_-TS samples. Figure 2 shows the FTIR spectra in the region of 4000 to 500 cm^−1^. In the AETS spectrum (Figure 2A), a broad absorption band can be observed in the region of 3300 cm^−1^, characteristic of molecular vibration and referring to the axial deformation of the hydroxyl group (–OH), which is typical in phenolic compounds. The bands between 1575 and 1360 cm^−1^ correspond to the stretching of the C=C group present in aromatic rings, followed by vibrations attributed to the C–O group of phenols, located between 1285 and 1078 cm^−1^, suggesting the presence of flavonoids and coumarins [21]. However, contributions from the bending vibration of adsorbed water (H–O–H), commonly detected in plant extracts and hydrated materials (AETS and Si-TS), cannot be excluded.

In the spectra of SiO_2_ and SiO_2_-TS, similar absorption bands were observed and can be attributed to typical vibrations of the silica network. The broad band in the region between 3511 and 3089 cm^−1^ corresponds to the O–H stretching vibrations of surface silanol groups and hydrogen-bonded hydroxyl species. The band observed around 1635 cm^−1^ is more likely associated with the bending vibration of molecularly adsorbed water (H–O–H), which is commonly detected in hydrated silica materials. In addition, the band around 1100–1060 cm^−1^ corresponds to the asymmetric stretching vibration of Si–O–Si bonds, which is characteristic of the silica network. The band observed near 955 cm^−1^ can be attributed to Si–O stretching vibrations associated with silanol groups or defective siloxane structures [20,22].

### 2.3. Behavioral Analysis

#### 2.3.1. Evaluation of Exploratory Behavior in the Open Field

**Figure 3:**
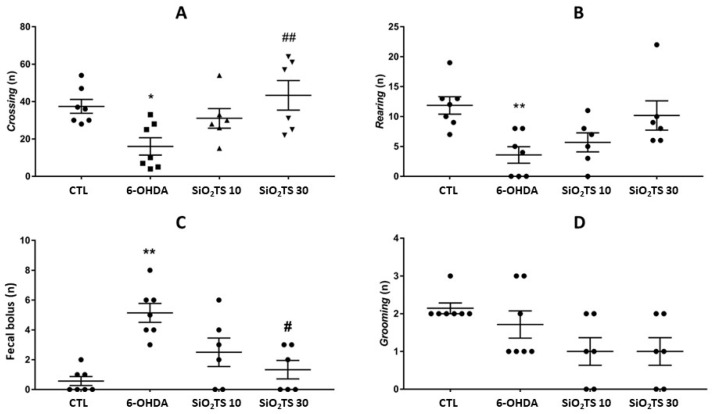
Open field test results for crossing (**A**), rearing (**B**), fecal boluses (**C**), and grooming (**D**) parameters. * Indicates a significant difference compared to the CTL group (*p* < 0.05), ** indicates a significant difference compared to the CTL group (*p* < 0.01), # indicates a significant difference compared to the 6-OHDA group (*p* < 0.05), ## indicates a significant difference compared to the 6-OHDA group (*p* < 0.01).

To analyze the crossing results (Figure 3A) in the open field test, a one-way ANOVA with Tukey’s post hoc test was performed [F(3, 22) = 3.861, *p* = 0.0219]. The animals lesioned with 6-OHDA showed a significant reduction in explorations of the apparatus compared to the control group (*p* = 0.0372). The animals in the SiO_2_-TS 10 (*p* = 0.8346) and SiO_2_-TS 30 (*p* = 0.8662) groups exhibited behavior similar to the control group, with no statistically significant difference between them. Additionally, a significant increase in crossings was observed in the SiO_2_-TS 30 group compared to the 6-OHDA group (*p* = 0.0085). The animals that received the toxin by intracerebral microinjection and were treated with silica particles (SiO_2_-TS 10 and SiO_2_-TS 30) showed statistically similar means (*p* = 0.4241).

Regarding rearing (Figure 3B), a Kruskal–Wallis analysis was performed with Dunn’s post hoc test (*p* = 0.0065). It was observed that the 6-OHDA animals presented a significant decrease compared to the control group (*p* = 0.0070). The lesioned animals treated with SiO_2_-TS exhibited behavior similar to the animals that did not receive the toxin (Control vs. SiO_2_-TS 10, *p* = 0.1078; Control vs. SiO_2_-TS 30, *p* > 0.9999). The same was true for the SiO_2_-TS groups when compared with each other (*p* > 0.9999) and between the SiO_2_-TS 10 (*p* > 0.9999) and SiO_2_-TS 30 (*p* = 0.2089) groups when compared with the 6-OHDA group.

Such motor deficits are caused by the induction of the animal model for studying Parkinson’s disease through the aforementioned toxin [23]. Additionally, animals lesioned with 6-OHDA that received daily treatment with SiO_2_-TS 30 mg/kg showed a significant increase in rearing, suggesting a neuroprotective action. When analyzing the mean elimination of fecal boluses (Figure 3C) during the open field test using Kruskal–Wallis with Dunn’s post hoc test (*p* = 0.0028), it was observed that the groups lesioned with the toxin and treated with SiO_2_-TS showed behavior similar to the control group (*p* > 0.9999). In contrast, animals in the 6-OHDA group (*p* = 0.0025) showed a significant increase in defecation. Animals in the SiO_2_-TS 30 group showed a significant reduction in fecal boluses compared to the 6-OHDA group (*p* = 0.0307). The SiO_2_-TS 10 group did not show any statistically significant difference when compared to the 6-OHDA group (*p* = 0.3476), nor to the SiO_2_-TS 30 group (*p* > 0.9999).

To analyze grooming (Figure 3D) in the open field test, a Kruskal–Wallis analysis with Dunn’s post hoc test was performed (*p* = 0.0551). No statistically significant difference was observed between any of the groups that received the intracerebral toxin compared to the group that did not receive the toxin (Control vs. 6-OHDA, *p* > 0.9999; Control vs. SiO_2_-TS 10, *p* = 0.1210; Control vs. SiO_2_-TS 30, *p* = 0.1210). Additionally, no significant increase was observed among the 6-OHDA lesioned groups when compared to each other (*p* > 0.9999).

#### 2.3.2. Contralateral Motor Symmetry

**Figure 4:**
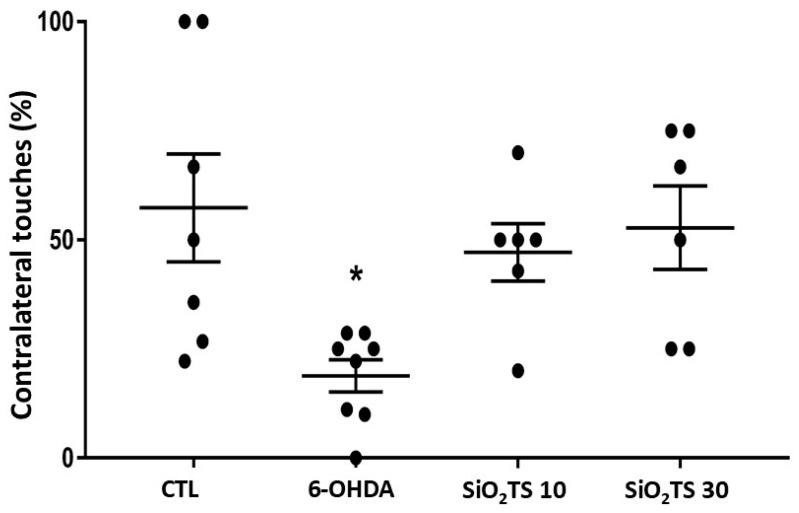
Results of the cylinder test, expressed as the percentage of contralateral touches. Kruskal–Wallis analysis with Dunn’s post-test was used to determine significance (*p* < 0.05). * Indicates a significant difference compared to the CTL group (*p* < 0.05).

In the cylinder test, spontaneous contralateral touches can serve as an indicator of brain injury severity. Regarding the mean percentages of contralateral touches (%CT), the control group exhibited a 51% rate, while the 6-OHDA group showed an 18.8% rate. The SiO_2_-TS 10 group displayed a 47.1% rate, and the SiO_2_-TS 30 group achieved a 52.8% rate (Figure 4).

In the cylinder test, spontaneous contralateral touches can serve as an indicator of brain injury severity. The limb contralateral to the injury site will be affected. Regarding the mean percentages of contralateral touches (%CT), the control group exhibited a 51% rate, while the 6-OHDA group showed an 18.8% rate. The SiO_2_-TS 10 group displayed a 47.1% rate, and the SiO_2_-TS 30 group achieved a 52.8% rate.

A non-parametric Kruskal–Wallis test, followed by Dunn’s post hoc analysis, revealed a significant difference in the number of contralateral touches between the control group and the 6-OHDA group [H(3) = 9.876, *p* = 0.0197]. However, post hoc comparisons did not show statistically significant differences between the control group and the SiO_2_-TS 10 or SiO_2_-TS 30 groups. Although there was a trend toward an increase in the number of contralateral touches in 6-OHDA-lesioned animals treated with silica particles compared to the vehicle group, this trend did not reach statistical significance. Moreover, there were no statistically significant differences between the groups treated with the different doses of silica particles.

#### 2.3.3. Immunohistochemical Analysis

**Figure 5:**
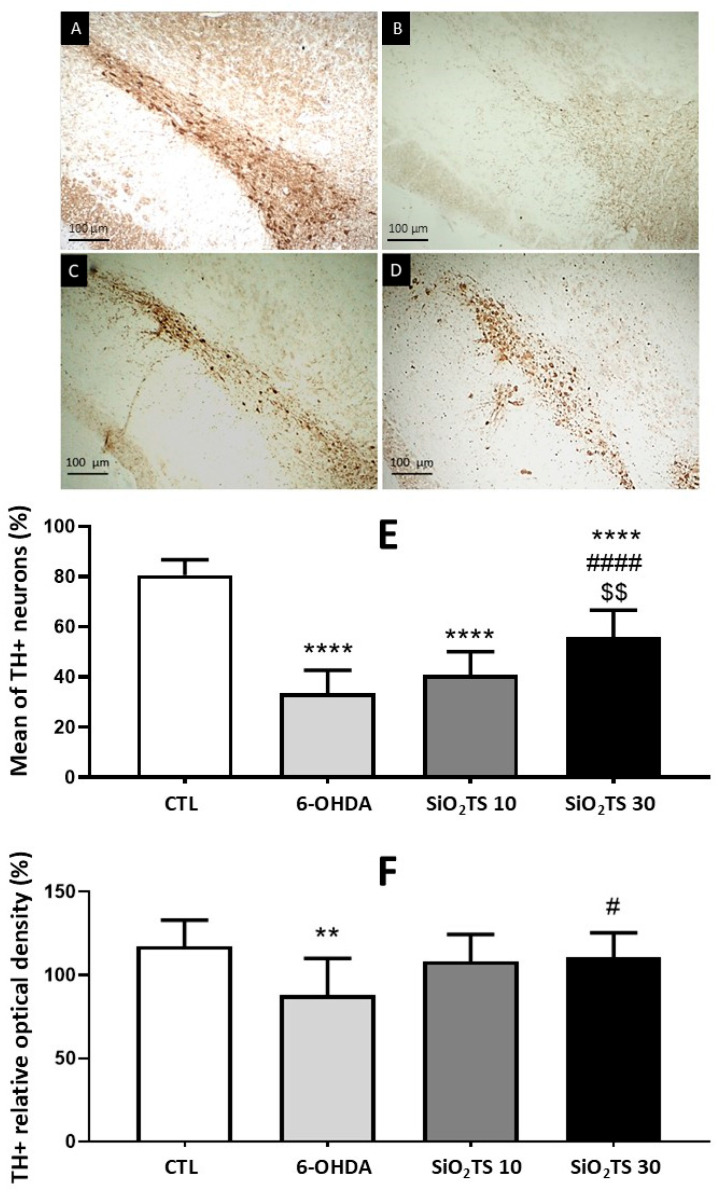
Photomicrographs (100×) of neurons immunoreactive for TH enzyme in the SNc of animals belonging to the control (**A**), 6-OHDA (**B**), SiO_2_-TS 10 (**C**), and SiO_2_-TS 30 (**D**) groups. TH-Positive Neuron Survival in the SNc (**E**). Striatal TH Relative Optical Density (**F**). ** indicates significant difference compared to the control (*p* < 0.01); **** indicates significant difference compared to the control (*p* < 0.0001); # indicates significant difference compared to 6-OHDA (*p* < 0.05); #### indicates significant difference compared to 6-OHDA (*p* < 0.0001); $$ indicates significant difference compared to SiO_2_-TS 10 (*p* < 0.01). Columns represent means, and bars represent standard deviations.

Based on the results in Figure 5, the immunohistochemical analysis of the SNc revealed a significant reduction in TH-positive neurons in all 6-OHDA-lesioned animals compared to the control group [F(3, 40) = 59.73, *p* < 0.0001]. Treatment with SiO_2_-TS 30 significantly increased the number of TH-positive neurons compared to the 6-OHDA group (*p* < 0.0001) and SiO_2_-TS 10 (*p* = 0.0016). No statistically significant differences were observed between the 6-OHDA and SiO_2_-TS 10 groups (*p* = 0.2310). However, there was a significant difference between the 6-OHDA and SiO_2_-TS 30 groups (*p* < 0.0001), as previously stated.

Results of the quantification of immunoreactive neurons and relative optical density of striatal fibers for the enzyme tyrosine hydroxylase (TH+) are shown concerning the contralateral side of the lesion (Figure 5E,F). A one-way ANOVA followed by Tukey’s post hoc test revealed a significant reduction in TH-positive striatal fibers in the 6-OHDA group compared to the control group [F(3, 36) = 5.256, *p* = 0.0041]. Both the SiO_2_-TS 10 and SiO_2_-TS 30 groups maintained striatal fiber densities similar to the control group, with no statistically significant differences. Moreover, SiO_2_-TS 30-treated animals showed a significant increase in TH-positive striatal fibers compared to the 6-OHDA group (*p* = 0.0295), while no significant differences were observed between the SiO_2_-TS groups (*p* = 0.9863).

#### 2.3.4. Astrocytic Expression in the Striatum Nucleus

**Figure 6:**
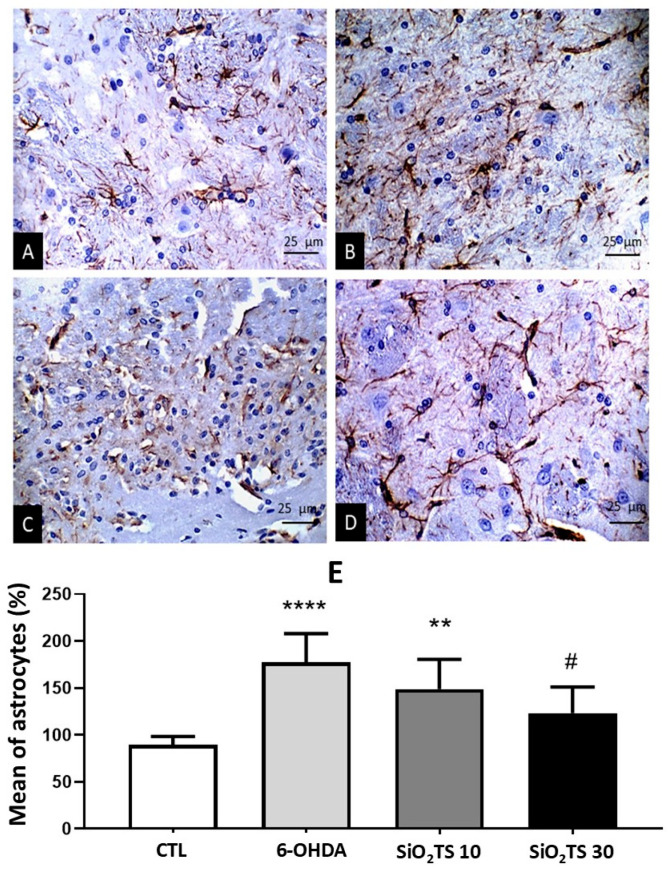
Quantification of immunoreactive astrocytes in the striatum nucleus, relative to the contralateral side of the lesion. Columns represent means, and bars represent standard deviations. Groups: (**A**) CTL; (**B**) 6-OHDA; (**C**) NPSiO2-TS 10 mg/kg; (**D**) NPSiO2-TS 30 mg/kg. (**E**) Striatal Astrocyte Quantification. ** indicates a significant difference compared to the control (*p* < 0.01). **** indicates a significant difference compared to the control (*p* < 0.0001). # indicates a significant difference compared to 6-OHDA (*p* < 0.05).

In Figure 6A–D, photomicrographs (400×) of the striatum nucleus demonstrate astrocytic expression in the respective groups. Kruskal–Wallis analysis with Dunn’s post hoc test (*p* < 0.0001) revealed a significant increase in astrocytic expression, as measured by GFAP optical density (Figure 6E), in the 6-OHDA (*p* < 0.0001) and SiO_2_-TS 10 (*p* = 0.0026) groups compared to the control group.

Animals treated with the highest dose (SiO_2_-TS 30) showed a reduction in astrocytic expression, similar to the control group (*p* = 0.1839) and different from the 6-OHDA group (*p* = 0.0313). There was no statistically significant difference between the SiO_2_-TS groups (*p* > 0.9999). Significant neuronal loss in the SNpc and dopaminergic fibers in the striatum nucleus was observed in animals induced with 6-OHDA and treated only with SiO_2_. This confirms the effectiveness of the 6-OHDA-induced nigrostriatal pathway lesion model, characterized by progressive dopaminergic neuron depletion over 28 days [24]. Concomitantly, 6-OHDA-injured animals exhibited an increased neuroinflammatory cascade, evidenced by elevated astrocytic expression persisting for at least 15 days post-surgery and potentially extending to 4 weeks [25].

## 3. Discussion

The extraction yield (21.9 ± 0.4%) obtained in this study was higher compared to the results of other authors for the *T. spathacea*, who reported yields of 2.8 to 8.37% for decoction [26,27], 17.9% for Soxhlet extraction [28], and 3.4% for rotary evaporation drying [29], all aimed at extracting bioactive compounds. Infusion is a conventional method effective in obtaining total phenolic compounds from plant material [30]. The AETS showed a TPC of 25.51 ± 2.39 mg GAE/g and a TFC of 6.10 ± 0.16 mg RE/g. These results corroborate the study by García-Varela et al. (2015) [31], which found 16.9 ± 3.7 mg/g of polyphenols under similar conditions (infusion of dried leaves). Furthermore, water as a solvent provides efficient phenolic compounds extraction from *T. spathacea* compared to other solvents such as methanol (1.5 ± 0.7 mg/g), ethanol (1.6 ± 0.2 mg/g), acetone (5.5 ± 1.1 mg/g), petroleum ether (1.4 ± 2.4 mg/g), chloroform (1.7 ± 2.5 mg/g), and hexane (0.7 ± 0.01 mg/g). Water poses minimal environmental risks and is regarded as a green solvent due to its safety, renewability, accessibility, and low cost. This solvent can dissolve numerous natural molecules, such as phenolic compounds from medicinal plants. In addition, many researchers consider water the greenest solvent in chemistry, both experimentally and industrially [32]. Comparing the total flavonoids obtained in AETS (6.10 ± 0.16 mg/g) to studies using the same plant material, it was observed that the quantity of these compounds was higher than in the methanolic extract obtained through ultrasound (0.7 mg/g) by Sánchez-Roque et al. (2017) [33] but lower than the amount extracted by Tan et al. (2014) [34] using 70% aqueous acetone and 1% formic acid (10.8 mg/g). This variation may be associated with the extraction methods, solvents, and the seasonality of the country where the plant material was collected [34].

The phytochemical profile of the *T. spathacea* extract employed in the synthesis of silica particles was characterized via LC-MS/MS, as previously established by Lopes et al. [14]. The analysis revealed a diverse chemical composition, primarily dominated by phenolic acids, including veratric acid, caffeic acid, p-coumaric acid, feruloyl threonic acid, and sinapic acid-O-hexoside. Furthermore, the extract contains flavonoids such as vicenin 2 and vestitol, alongside the coumarin fraxetin and the iridoid deacetylasperuloside. Other bioactive constituents identified include helonioside A, pinellic acid isomers, and several fatty acid derivatives, in addition to three compounds classified as unknown.

These metabolites, particularly the polyphenolic compounds, are instrumental in the nucleation, stabilization, structural formation, and bioactivity of the silica particles of this present study, likely through interactions mediated by their functional hydroxyl groups. Meanwhile, iridoids, phenylpropanoid glycosides, and fatty acid derivatives contribute additional chemical diversity, potentially influencing antioxidant capacity, hydrophobic interactions, and particle morphology.

The SiO_2_-TS sample exhibited greater stability than AETS, indicating that silica was a suitable choice for enhancing AETS stability. These results corroborate the study by Ciobanu et al. (2019) [17], which also found greater thermal decomposition in pure extracts (42 and 48%) compared to extracts associated with mesoporous silica, where the TG curves showed a mass loss of 21 to 41%. Consistent with the literature, biocomposites derived from plant material (orange peel) and carried by silica exhibited similar thermal degradation patterns to the present study. Degradation typically began around 50 °C due to the presence of an aqueous extract [35].

The open field test, in addition to enabling functional assessment, allows for the measurement of anxious behaviors in animals, with the number of fecal boluses being a parameter to assess anxiety [14,23]. Thus, the significant increase in defecation observed in the 6-OHDA group compared to the control group indicates a higher level of anxiety due to the lesion. It is suggested that SiO_2_-TS at 30 mg/kg had an anxiolytic effect, as evidenced by the significant reduction in fecal boluses in the 6-OHDA group.

Lopes et al. (2024) [14] reported that AETS at a dose of 100 mg/kg had a sedative effect (decrease in anxiety parameters and motor activity) when compared to the 6-OHDA group. The anxiolytic activity may be associated with the presence of fraxetin, a coumarin, which, in addition to acting through an anti-inflammatory mechanism by mediating the expression of interleukins (IL-1β, IL-6), TNF-α, nitric oxide, and microglia, is associated with the attenuation of anxious and depressive behavior in vivo [36,37]. In the present study, a decrease in the anxiety parameter (fecal boluses) was observed without compromising spontaneous motor activity. Another biocompound that may be associated with the reduction in fecal boluses in this research is vicenin-2, a flavonoid present in *T. spathacea* and predominant in Passiflora edulis, which has been linked to antioxidant [37] and antidepressant actions, acting in synergy with other compounds [38].

Animals with 6-OHDA-induced lesions and treated daily with SiO_2_-TS at 10 and 30 mg/kg demonstrated significant recovery of neurons in the SNc, preservation of TH+ positive fibers, and reduced astrocytic expression in the striatum nucleus. Lopes et al. (2024) [14] reported similar findings with AETS but observed a sedative effect at higher doses. These results suggest that the mechanism of action of SiO_2_-TS against 6-OHDA-induced nigrostriatal pathway injury involves anti-neuroinflammatory activity. This may be attributed to the biocompounds present in its chemical composition [14].

Among the phenolic acids previously identified by the AETS research group, caffeic acid and p-coumaric acid were related to the protection of neuronal cells (PC-12) against cytotoxicity and prevention of apoptosis by oxidative stress [39]. Caffeic acid acts by reducing astrocytic expression and inflammatory mediators, in addition to protecting dopaminergic neurons and motor deficits [40]. In an in vivo model for studying Parkinson’s disease induced by rotenone and treated with caffeic acid, the animals showed attenuation of motor deficits, in addition to lower microglial expression and inflammatory mediators, such as inducible nitric oxide synthase (iNOS), cyclooxygenase-2 (COX-2), and nuclear factor kappa B (NFκB) [41].

Veratric acid, a phenolic acid also identified in AETS, acts with an anti-inflammatory mechanism, regulating the expression of IL-6 and nitric oxide, increasing endogenous antioxidants such as SOD, CAT, and GSH, consequently attenuating oxidative stress and apoptosis [42,43,44]. Vestitol modulates NF-κB activation, consequently acting on the expression of interleukins (IL-1β and IL-1α) [45].

The bioactive compounds in AETS may have mitigated potential cellular events associated with silica lung toxicity, such as cell apoptosis and increased levels of TNF-α, IL-1αβ, and IL-6. While the literature primarily focuses on the in vivo toxicity of crystalline silica, intranasal exposure to high doses (2.6 to 27 mg/m^3^) of amorphous silica over extended periods (28 to 90 days) can still lead to inflammatory responses, cell death, and pulmonary fibrosis [46]. Despite the 15-day treatment duration and lower doses (10 and 30 mg/kg), more targeted studies are needed to fully assess the effects of *T. spathacea* on silica-induced lung toxicity. This is a limitation of the current research.

## 4. Materials and Methods

### 4.1. Drugs and Chemicals

Gallic acid monohydrate PA from Neon Comercial Reagentes Analíticos Ltda (São Paulo, Brazil), rutin hydrate (≥94%), ascorbic acid, and Folin–Ciocalteu reagent from Dinâmica Química Contemporânea^®^ (Indaiatuba, Brazil) were used in the study. Apomorphine, 3,3′-diaminobenzidine (DAB), primary antibodies, and 6-hydroxydopamine (6-OHDA) were obtained from Sigma-Aldrich^®^ (St. Louis, MO, USA). Antibodies and the peroxidase-labeled streptavidin LSAB2 kit were supplied by DAKO^®^ (Carpinteria, CA, USA). Veterinary pentabiotic was acquired from Fort Dodge^®^ (Campinas, Brazil), while ketamine and xylazine were obtained from Syntec^®^ (São Paulo, Brazil). Hematoxylin was sourced from Merck^®^ (Darmstadt, Germany).

### 4.2. Plant Material and Extraction Process

Leaves of *T. spathacea* were collected in Aracaju, Sergipe, Brazil. After a 48-h drying period in a hot-air oven at 45 °C, the samples were ground to a particle size of 1.18–0.50 mm using Tyler series sieves. The milled material was stored at room temperature, protected from light. The aqueous extract of *T. spathacea* (AETS) was prepared in triplicate by infusion, following the methodology described by Lopes et al. (2024) [14]. The infusion was performed using 1 g of sample in 30 mL of distilled water, previously heated to 100 °C. The heated water was added to the weighed biomass and allowed to stand for 15 min without additional heating. Subsequently, the extract was filtered through a microporous filter, and the filtrate was collected in pre-weighed glass containers. To facilitate solvent evaporation, the extract was placed in an oven at 45 °C. The yield of the extraction process was determined as previously described by Lopes et al. (2024) [14].

#### Total Phenolic and Flavonoid Content

The total phenolic content (TPC) was quantified using the Folin–Ciocalteu assay, with absorbance recorded at 765 nm. Results were expressed as milligrams of gallic acid equivalents per gram of dry extract (mg GAE/g). In parallel, the total flavonoid content (TFC) was determined by the aluminum nitrate colorimetric method at 425 nm, employing rutin as the calibration standard. Data were expressed as milligrams of rutin equivalents per gram of dry extract (mg RE/g). Both determinations were conducted in accordance with the protocol established by Lopes et al. (2024) [14].

### 4.3. Development and Characterization of T. spathacea-Loaded Silica

#### 4.3.1. Silica Synthesis Methods

The synthesis of silica particles (SiO_2_) and *T. spathacea*-loaded silica particles (SiO_2_-TS) was performed with adaptations based on the procedure described by Periakaruppan et al. (2022) [22]. The synthesis of both particle types involved the addition of 6 mL of tetraethoxysilane (TEOS) as the silica precursor. For the preparation of SiO_2_-TS, 9 mL of AETS (aqueous extract of *T. spathacea)* diluted in distilled water (15 mg/mL) were added to the reaction mixture, providing water for the hydrolysis reaction. The resulting molar ratio of H_2_O:TEOS was approximately 18.5:1. The reaction mixture was stirred for 10 min, followed by the addition of 3 mL of hydrochloric acid solution (HCl, 1 M), corresponding to an HCl:TEOS molar ratio of approximately 0.11:1. The system was then stirred for an additional 15 min at room temperature, after which gel formation was observed.

This gel was then placed in an air circulation oven and maintained at 40 °C for 12 h. Subsequently, the oven temperature was increased to 60 °C and held for an additional 24 h. After maceration, the final powder samples exhibited distinct coloration: SiO_2_ appeared white, whereas SiO_2_-TS presented a brownish tone, indicative of the presence of bioactive compounds. The resulting powders were stored in airtight containers for further analysis.

#### 4.3.2. Thermogravimetric Analysis (TGA)

Thermal stability and composition assessments were conducted using an STA7200RV thermogravimetric analyzer (Hitachi, Chiyoda City, Japan). The analysis was performed over a temperature range of 25 to 1000 °C, under a nitrogen flow rate of 50 mL/min. The samples analyzed included AETS, SiO_2_, and SiO_2_-TS, enabling comparative evaluation of their thermal properties.

#### 4.3.3. Fourier Transform Infrared Spectroscopy (FTIR)

To confirm the presence of AETS within the silica particles through functional group vibrations, Fourier Transform Infrared Spectroscopy (FTIR) analysis was performed on AETS, SiO_2_, and SiO_2_-TS. The spectra were recorded using an attenuated total reflectance (ATR) spectrometer (Invenio R, Bruker, Billerica, MA, USA). Infrared absorption measurements were conducted in the range of 4000 to 600 cm^−1^, with 16 scans per sample and a maximum resolution of 4 cm^−1^, ensuring high spectral accuracy for detecting molecular interactions.

### 4.4. Biological Assay

#### 4.4.1. Animals

The study utilized adult male Wistar rats, weighing between 180 and 250 g. The animals were housed under controlled environmental conditions, with a temperature maintained at 21 ± 2 °C and a 12-h light/dark cycle. They had unrestricted access to standard rat chow (Labina^®^ Purina, São Paulo, Brazil) and water [14]. All experimental procedures were approved by the Animal Experimentation Ethics Committee of Tiradentes University on 9 October 2019 (protocol no. 020919). The study followed the ethical guidelines of the Brazilian National Council for the Control of Animal Experimentation (CONCEA) and was conducted between 1 December 2019, and 31 December 2021. The experimental design prioritized minimizing animal suffering and adhered to the principles of reducing the number of animals used while ensuring scientific validity.

#### 4.4.2. Experimental Design

All groups underwent stereotactic surgery, with intracerebral inductions performed via intrastriatal microinjection of 6-hydroxydopamine (6-OHDA) or 0.9% saline solution. Treatment with silica particles was administered intranasally (30 µL/day) for 15 consecutive days (see Table 1 for group details). The formulations of *T. spathacea*-loaded silica were prepared by incorporating AETS (10 or 30 mg/kg), based on previous studies demonstrating AETS’s neuroprotective effects when administered orally at 10, 30, or 100 mg/kg for 30 days, with no signs of acute renal or hepatic toxicity [14].

#### 4.4.3. Unilateral 6-OHDA Lesions

Initially, animals were weighed and then anesthetized via intraperitoneal injection of ketamine hydrochloride (10 mg/kg) and xylazine hydrochloride (100 mg/kg) at a volume of 0.05 mL per 100 g of body weight. Stereotaxic surgery followed the methodology of Lopes et al. (2024) [14]. Using Bregma as the reference point (AP +1.0 mm, ML +3.0 mm, DV -5.0 mm), a Hamilton micro-syringe (10 μL) connected to an automatic infusion pump (Insight, São Paulo, Brazil) delivered 3 μL of 6-hydroxydopamine (6-OHDA) (Sigma-Aldrich, São Paulo, Brazil) in 0.9% saline containing 0.02% ascorbic acid (Sigma-Aldrich, Brazil) at 1 μL/min into the right striatum. To prevent reflux, the cannula connected via polyethylene tubing (PE 10) remained in place for 5 min post-injection. The control group received an equivalent volume of saline containing 0.02% ascorbic acid instead of 6-OHDA, following the same procedure. Post-surgery, incisions were closed with polyglactin 910, 5-0 sutures (PolySuture^®^, São Sebastião do Paraíso, Brazil), and animals received an intramuscular injection of veterinary pentabiotic for small animals (Forte Dodge Saúde Animal LTDA, Campinas, Brazil, 0.2 mL/kg). Animals were kept warm under a 60 W lamp until recovery from anesthesia [14].

#### 4.4.4. Open Field Test

The evaluation of the open field test was conducted in a square wooden arena with sides measuring 35 cm in height, and the base subdivided into 16 quadrants. The arena was cleaned with 20% alcohol between each test to prevent the scent of the previous animal from influencing the next one. The test was performed on all animals individually on the 10th day after the injury and lasted for 5 min [14].

#### 4.4.5. Cylinder Test

The test was conducted in a transparent cylinder measuring 30 cm in height and 16 cm in diameter. Two mirrors were positioned behind the cylinder to allow a 360° view. The evaluation occurred on the 14th day post-surgery, with each test lasting 5 min and recorded on video. Contacts with the cylinder walls were quantified when made with the contralateral or ipsilateral forelimb, with simultaneous touches excluded from the count [47].

#### 4.4.6. Immunohistochemistry

Slides with brain sections were subjected to deparaffinization, dehydration, washing with phosphate-buffered saline (PBS) 0.1 M, pH 7.4, and antigen retrieval in a steamer with citrate buffer (pH 6.0) in 3 cycles of 5 min each. Endogenous peroxidase blocking (1% H_2_O_2_ in PBS) was then performed for 7 min. The immunohistochemistry procedures used the following primary antibodies: (1) anti-tyrosine hydroxylase (TH, marker for dopaminergic neurons, produced in mice, at a dilution of 1:500, clone TH-16, batch no. T2928, Sigma Aldrich Chemical Company, St. Louis, MO, USA) and (2) monoclonal anti-glial fibrillary acidic protein (GFAP, marker for astrocytes, produced in mice, clone S206A-8, 1:100, batch no. SAB5201104, Sigma Aldrich Chemical Company, St. Louis, MO, USA). The primary antibodies were prepared for incubation with the primary antibody diluent (Sigma-Aldrich, St. Louis, MO, USA) for 2 h at room temperature. Subsequently, incubation with the secondary antibody (DAKO LSAB2 kit, DAKO Corp., Carpinteria, CA, USA), conjugated with a streptavidin-peroxidase complex (DAKO Corp., Carpinteria, CA, USA) was performed. This was done in sequence: biotinylated for 30 min, followed by streptavidin for another 30 min. For visualization, sections were incubated with 3,3′-diaminobenzidine tetrahydrochloride (DAB kit, Vector Laboratories, Newark, CA, USA), diluted in Tris-buffered saline (TBS, pH 7.4) with 0.3% H_2_O_2_, and washed in distilled water. The slides were dehydrated, cleared, and coverslips were mounted with Canada balsam. Counterstaining with Harris hematoxylin was applied for 3 min on GFAP slides [48].

#### 4.4.7. Relative Optical Density for TH

Slides from immunohistochemistry for TH were analyzed. Images were captured using an Olympus CX31 microscope (Hachioji, Japan) with an Olympus video camera. Relative optical density was measured with ImageJ software, version 1.52 (National Institutes of Health, Bethesda, MD, USA), using a grayscale range from 0 (darkest, highest intensity) to 255. Quadrants of the striatal nucleus (dorsomedial, dorsolateral, and ventrolateral) were evaluated, each with an area of 480 μm^2^. Average areas were normalized to the cortex, serving as the control area. Results were expressed as the percentage of optical density on the ipsilateral side compared to the contralateral side. The best detail can be observed in Lopez et al. (2024) [14].

### 4.5. Statistical Analysis

The data were analyzed for normality using the Shapiro–Wilk test. Normally distributed samples underwent one-way ANOVA with Tukey’s post hoc test. Non-normally distributed samples were analyzed using the Kruskal–Wallis test with Dunn’s post hoc test. Significance was set at *p* < 0.05. GraphPad Prism version 8.0 was used.

## 5. Conclusions

The infusion method proved to be effective for extracting neuroprotective compounds from *T. spathacea* leaves. This was evidenced by the satisfactory yield of total phenolic and flavonoid compounds compared to literature values. Additionally, the presence of carboxyl and hydroxyl groups in SiO_2_-TS, along with silanol and siloxane groups, confirmed the successful loading of AETS extract. Mesoporous silica enhanced the thermal stability of AETS, as demonstrated by the smaller mass loss of SiO_2_-TS compared to AETS, suggesting reduced degradation of bioactive compounds. In vivo evaluation revealed functional improvement in behavioral tests following nigrostriatal pathway lesions in the SiO_2_-TS 30 mg/kg group, indicating a neuroprotective effect of the SiO_2_-TS. Moreover, this dose demonstrated anxiolytic effects without sedation. Treatment with SiO_2_-TS 30 mg/kg protected dopaminergic neurons, preserved striatal fibers, and attenuated astrocytic expression. Therefore, mesoporous silica particles carrying *T. spathacea* at a dose of 30 mg/kg exhibited a neuroprotective effect against nigrostriatal pathway lesions in this preclinical Parkinson’s disease model.

## 6. Patents

The work described in this manuscript resulted in patent BR 10 2024 020061 6.

## Figures and Tables

**Table 1:** Experimental design and treatment groups (*n* = 7 per group).

Experimental Group	Intrastriatal Induction	Treatment	Dosage (mg/kg) EATS
Control	0.9% Saline	SiO_2_ (Vehicle)	___
6-OHDA	6-Hydroxydopamine	SiO_2_ (Vehicle)	___
SiO_2_-TS 10	6-Hydroxydopamine	SiO_2_-TS	10
SiO_2_-TS 30	6-Hydroxydopamine	SiO_2_-TS	30

## Data Availability

The original contributions presented in this work are fully documented in this manuscript. All further inquiries should be addressed to the corresponding author.

## Abbreviations

The following abbreviations are used in this manuscript:

PD	Parkinson’s disease
SNpc	Substantia nigra pars compacta
ROS	Reactive oxygen species
AETS	Aqueous Extract of *T. spathacea*
SiO_2_	Silica Particles
SiO_2_-TS	*T. spathacea*-Loaded Silica Particles
HCl	Hydrochloric Acid Solution
TGA	Thermogravimetric Analysis
FTIR	Fourier Transform Infrared Spectroscopy
ATR	Attenuated Total Reflectance
CONCEA	Control of Animal Experimentation
6-OHDA	6-Hydroxydopamine
SiO_2_-TS 10	Treatment with SiO_2_-TS (10 mg/kg)
SiO_2_-TS 30	Treatment with SiO_2_-TS (30 mg/kg)
PBS	Phosphate-Buffered Saline
TH	Anti-Tyrosine Hydroxylase
GFAP	Anti-Glial Fibrillary Acidic Protein
TBS	Tris-Buffered Saline
TPC	Total Phenolic Content
TFC	Total Flavonoid Content
–OH	Hydroxyl Group
Si–OH	Silanol
Si–O–Si	Siloxane
%CT	Contralateral Touches
PC-12	Neuronal Cells
iNOS	Inducible Nitric Oxide Synthase
